# Supplementary material for: A recombined Sr26 and Sr61 disease resistance gene stack in wheat encodes unrelated NLR genes
Source: Nat Commun. 2021 Jun 7;12:3378. doi: 10.1038/s41467-021-23738-0 (PMC8184838; doi:10.1038/s41467-021-23738-0)
Supplement: Supplementary file 1 — Supplementary information [file 41467_2021_23738_MOESM1_ESM.pdf]

A recombined *Sr26* and *Sr61* disease resistance gene stack in wheat encode unrelated *NLR* genes

Zhang *et al.*

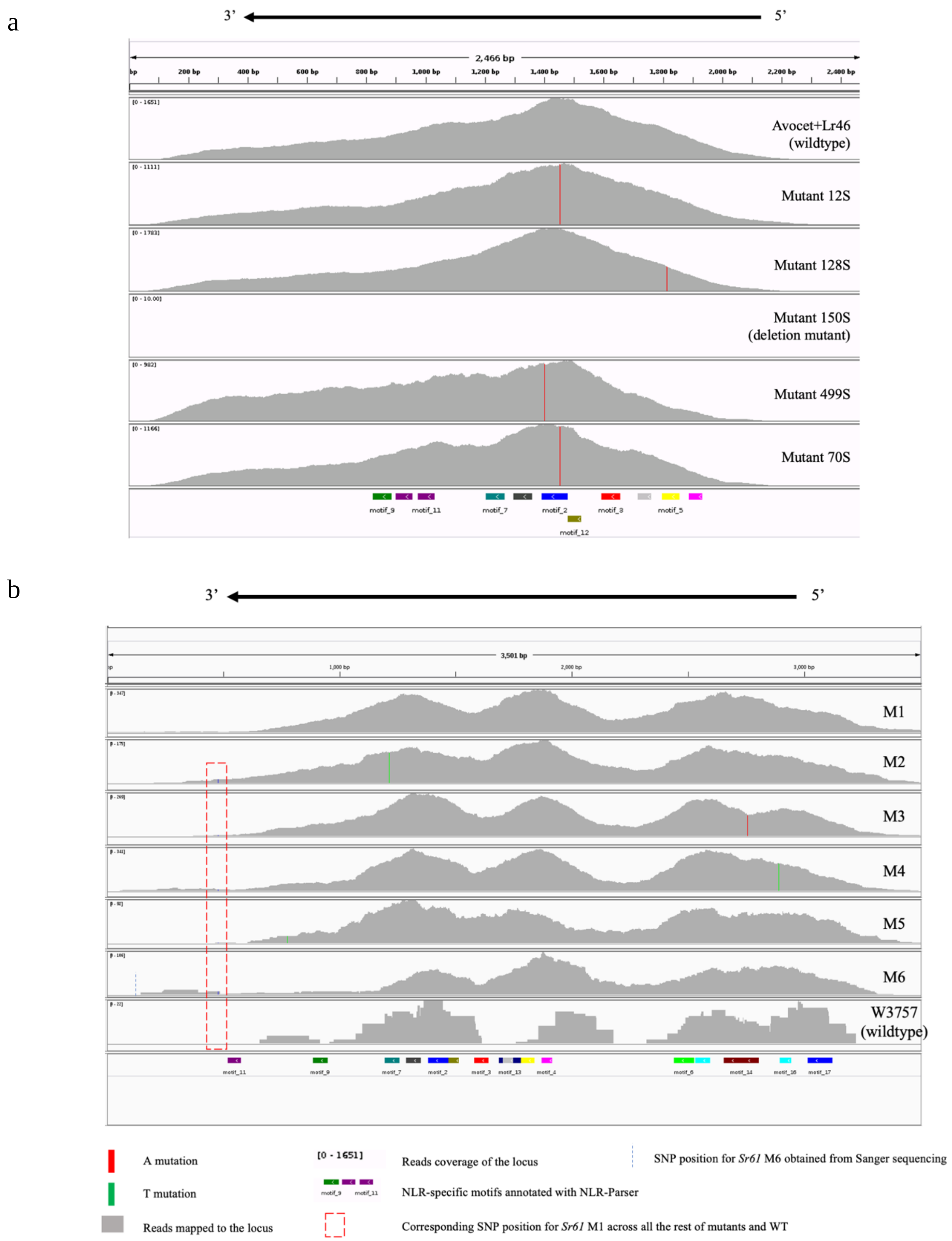

Supplementary Figure 1. IGV analysis showing the identification of mutations in *Sr26* and *Sr61* susceptible mutants using MutRenSeq.
a. IGV output indicating SNP changes in mutants used for identifying the *Sr26* gene. The image illustrates sequence read numbers (Y-axis, read number range shown in the top left corner i.e. 0-1651 for Avocet+Lr46) and position in the *Sr26* candidate gene contigs (X-axis). The gene orientation is shown in 3’-5’ direction. The wild-type Avocet+Lr46 *de novo* sequence assembly is shown at the top with equivalent mutant sequences below. Four susceptible mutants contain SNPs at positions indicated with a red line (all G-A transitions). One mutation was a deletion and hence no sequence was recovered. Mutants 12S and 70S possess an identical SNP change and hence are likely sibs. Coloured rectangles represent conserved NLR motifs identified by NLR-Parser. b. IGV analysis as described above for the *Sr61* locus with five of six susceptible mutants carrying a mutation in the candidate contig as identified by MutRenSeq. The SNP mutation in M6 was identified from whole gene sequence alignment by Sanger sequencing rather than MutRenSeq and the SNP position in this mutant is indicated as a dotted blue line. The complete locus was *de novo* assembled using the sequence from mutant M1. The SNP unique to M1 and therefore present in the remaining mutants is highlighted in a dotted orange frame. The position of A and T mutations are shown as orange and green lines, respectively, in each mutant sequence.

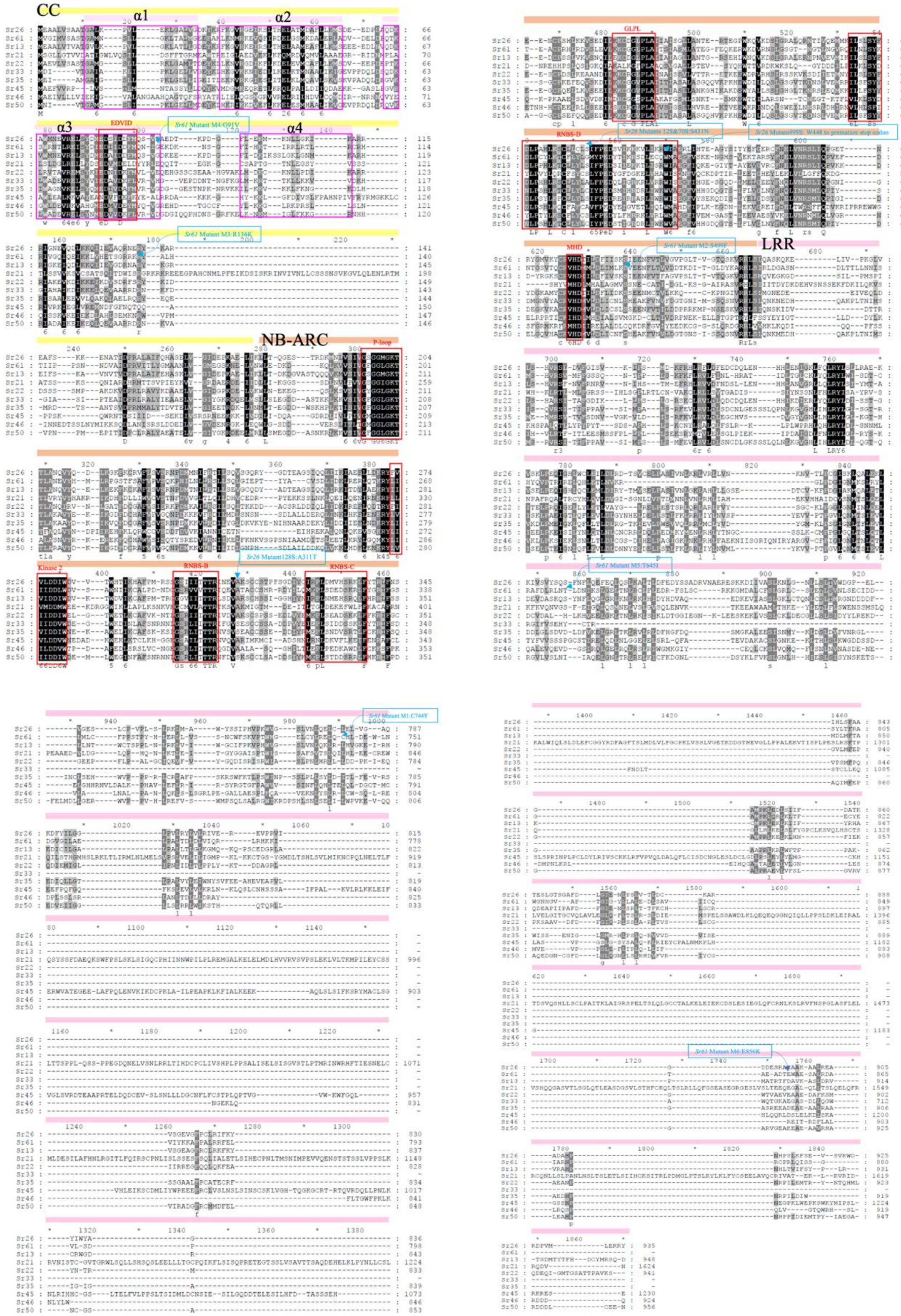

Supplementary Figure 2. Alignment of proteins encoded by cloned wheat *Sr* genes.

Alignment of Sr13, Sr21, Sr22, Sr26, Sr35, Sr33, Sr45, Sr46, Sr50 and Sr61 proteins. The CC (coiled-coil), NB (nucleotide binding)-ARC, and LRR (leucine-rich-repeat) domains are indicated by yellow, peach, and pink bars above, respectively. The conserved motifs (EDVID, P-loop, Kinase 2, RNBS-B, RNBS-C, GLPL, RNBS-D, and MHD) are boxed in red and labelled below the alignment. Four α-helices based on the Sr33 CC domain structure are boxed in purple. Amino acids indicated by blue arrows and blue boxes show the positions of aa changes in the Sr26 and Sr61 mutants.

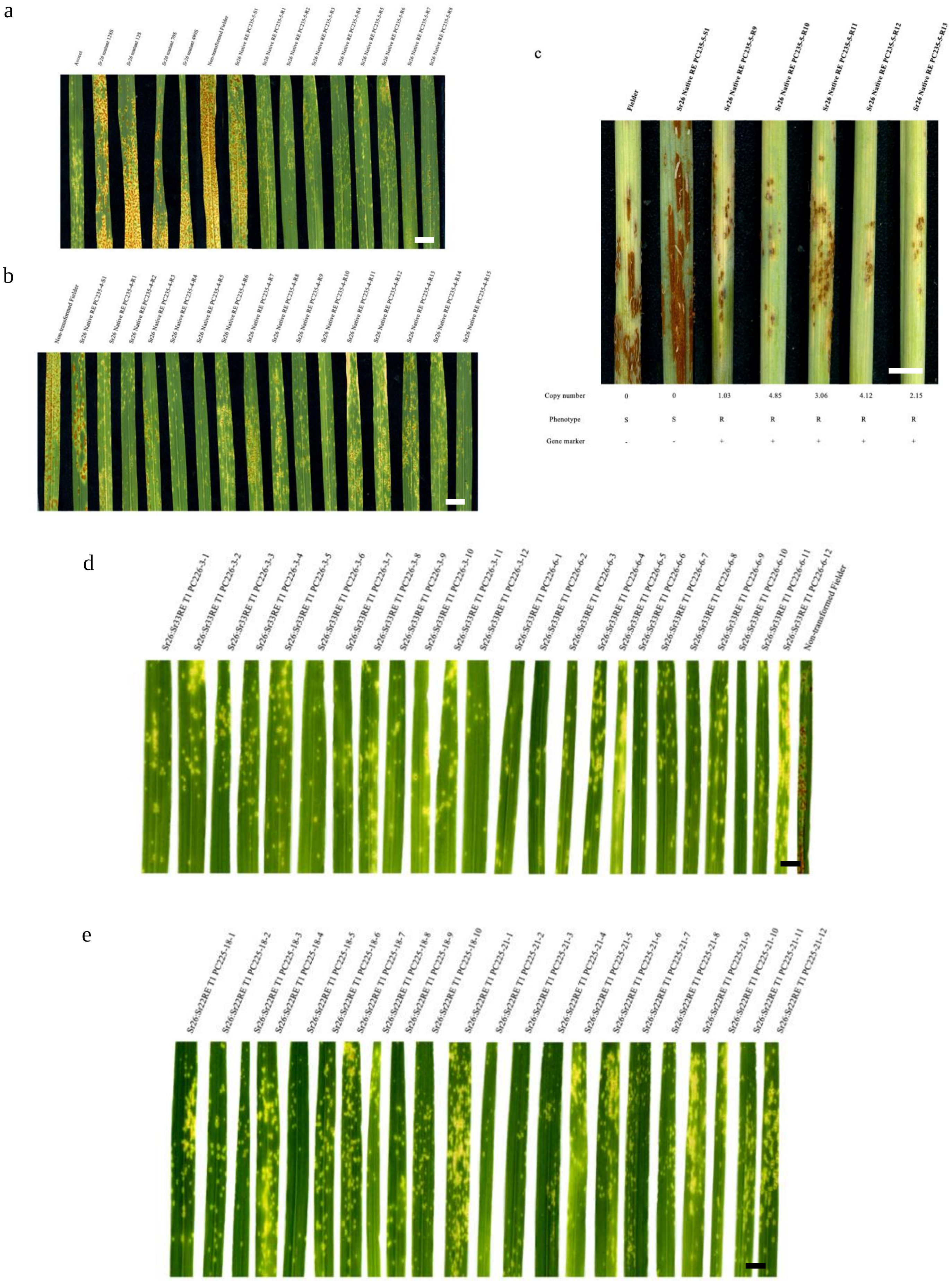
a
Avocet
Sr26 mutant 128S
Sr26 mutant 12S
Sr26 mutant 70S
Sr26 mutant 499S
Non-transformed Fielder
Sr26 Native RE PC235-5-S1
Sr26 Native RE PC235-5-R1
Sr26 Native RE PC235-5-R2
Sr26 Native RE PC235-5-R3
Sr26 Native RE PC235-5-R4
Sr26 Native RE PC235-5-R5
Sr26 Native RE PC235-5-R6
Sr26 Native RE PC235-5-R7
Sr26 Native RE PC235-5-R8
b
Non-transformed Fielder
Sr26 Native RE PC235-4-S1
Sr26 Native RE PC235-4-R1
Sr26 Native RE PC235-4-R2
Sr26 Native RE PC235-4-R3
Sr26 Native RE PC235-4-R4
Sr26 Native RE PC235-4-R5
Sr26 Native RE PC235-4-R6
Sr26 Native RE PC235-4-R7
Sr26 Native RE PC235-4-R8
Sr26 Native RE PC235-4-R9
Sr26 Native RE PC235-4-R10
Sr26 Native RE PC235-4-R11
Sr26 Native RE PC235-4-R12
Sr26 Native RE PC235-4-R13
Sr26 Native RE PC235-4-R14
Sr26 Native RE PC235-4-R15
c
Fielder
Sr26 Native RE PC235-5-S1
Sr26 Native RE PC235-5-R9
Sr26 Native RE PC235-5-R10
Sr26 Native RE PC235-5-R11
Sr26 Native RE PC235-5-R12
Sr26 Native RE PC235-5-R13
Copy number 0 0 1.03 4.85 3.06 4.12 2.15
Phenotype S S R R R R R
Gene marker - - + + + + +
d
Sr26:Sr33RE T1 PC226-3-1
Sr26:Sr33RE T1 PC226-3-2
Sr26:Sr33RE T1 PC226-3-3
Sr26:Sr33RE T1 PC226-3-4
Sr26:Sr33RE T1 PC226-3-5
Sr26:Sr33RE T1 PC226-3-6
Sr26:Sr33RE T1 PC226-3-7
Sr26:Sr33RE T1 PC226-3-8
Sr26:Sr33RE T1 PC226-3-9
Sr26:Sr33RE T1 PC226-3-10
Sr26:Sr33RE T1 PC226-3-11
Sr26:Sr33RE T1 PC226-3-12
Sr26:Sr33RE T1 PC226-6-1
Sr26:Sr33RE T1 PC226-6-2
Sr26:Sr33RE T1 PC226-6-3
Sr26:Sr33RE T1 PC226-6-4
Sr26:Sr33RE T1 PC226-6-5
Sr26:Sr33RE T1 PC226-6-6
Sr26:Sr33RE T1 PC226-6-7
Sr26:Sr33RE T1 PC226-6-8
Sr26:Sr33RE T1 PC226-6-9
Sr26:Sr33RE T1 PC226-6-10
Sr26:Sr33RE T1 PC226-6-11
Sr26:Sr33RE T1 PC226-6-12
Non-transformed Fielder
e
Sr26:Sr22RE T1 PC225-18-1
Sr26:Sr22RE T1 PC225-18-2
Sr26:Sr22RE T1 PC225-18-3
Sr26:Sr22RE T1 PC225-18-4
Sr26:Sr22RE T1 PC225-18-5
Sr26:Sr22RE T1 PC225-18-6
Sr26:Sr22RE T1 PC225-18-7
Sr26:Sr22RE T1 PC225-18-8
Sr26:Sr22RE T1 PC225-18-9
Sr26:Sr22RE T1 PC225-18-10
Sr26:Sr22RE T1 PC225-21-1
Sr26:Sr22RE T1 PC225-21-2
Sr26:Sr22RE T1 PC225-21-3
Sr26:Sr22RE T1 PC225-21-4
Sr26:Sr22RE T1 PC225-21-5
Sr26:Sr22RE T1 PC225-21-6
Sr26:Sr22RE T1 PC225-21-7
Sr26:Sr22RE T1 PC225-21-8
Sr26:Sr22RE T1 PC225-21-9
Sr26:Sr22RE T1 PC225-21-10
Sr26:Sr22RE T1 PC225-21-11
Sr26:Sr22RE T1 PC225-21-12

f

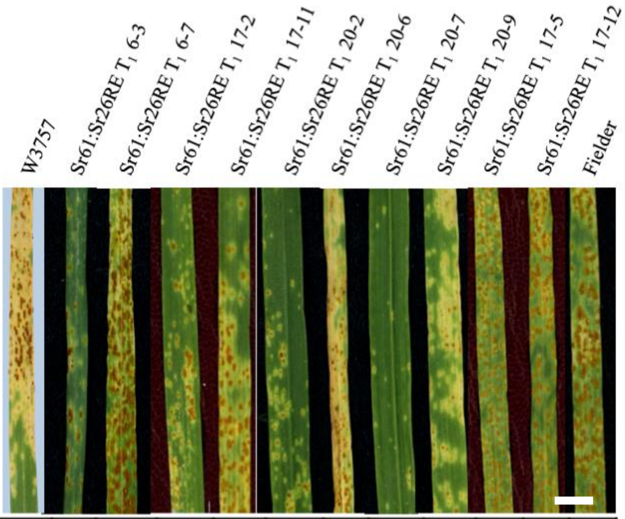

Sr61 gene marker	+	+	+	+	+	+	+	+	-	-	-
Copy number	3.73	4.97	2.99	0.99	/	12.5	7.5	/	0	0	0

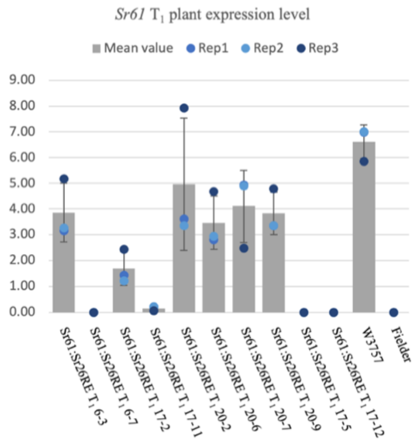

Supplementary Figure 3. Validation of *Sr26* and *Sr61* candidate genes by transformation.
a-b. Phenotypic responses of Avocet (proxy for Avovet+Lr46, *Sr26* mutants, susceptible Fielder control and *Sr26:NativeRE* T_1 plants from transgenic T_0 plant groups PC235-5 (a), PC235-4 (b). Plants were inoculated at the two-leaf stage with *Pgt* race 98-1,2,3,5,6+Sr50 and photographed 12 days post inoculation. Bar shows 1 cm.
c. Segregation and copy number of the *Sr26:NativeRE* transgene in T_1 progeny from group PC235-5. Reactions to *Pgt* race 98-1,2,3,5,6+Sr50 are shown on column from each plant (above) and transgene presence and copy number, determined using digital droplet PCR, are indicated below. Bar shows 1 cm.
d. T_1 families from T_0 plant groups PC225-18 and PC225-21 containing the *Sr26:Sr22RE* transgene. T_1 plants and susceptible Fielder controls were infected with *Pgt* race 98-1,2,3,5,6+Sr50. Bar shows 1 cm.
e. T_1 families from T_0 plant groups PC226-3 and PC226-6 containing the *Sr26:Sr33RE* transgene. T_1 plants and susceptible Fielder controls were infected with *Pgt* race 98-1,2,3,5,6+Sr50. Bar shows 1 cm.
f. Infection with *Pgt* race 98-1,2,3,5,6+Sr50 of T_1 plants segregating for the Sr61:Sr26RE transgene, the resistant (W3757) and susceptible (Fielder) control lines (left panel). T_1 plants were derived from T_0 transgenic lines *Sr61:Sr26RE-6*, *Sr61:Sr26RE-17*, *Sr61:Sr26RE-20*. The presence and copy number of the transgene in each line is shown below the image. Expression levels of the *Sr61:Sr26RE* transgene in T_1 plants and endogenous *Sr61* gene in line W3757 12 days post infection with *Pgt* race 98-1,2,3,5,6+Sr50 are shown at the right panel. Data are presented as mean values +/- SD and each experiment was performed in 3 biological and 3 technical repeats. Each set of the repeats was represented by dot points with same color. Bar shows 1 cm. Source data underlying Supplementary Figure 3f are provided as Source Data file.

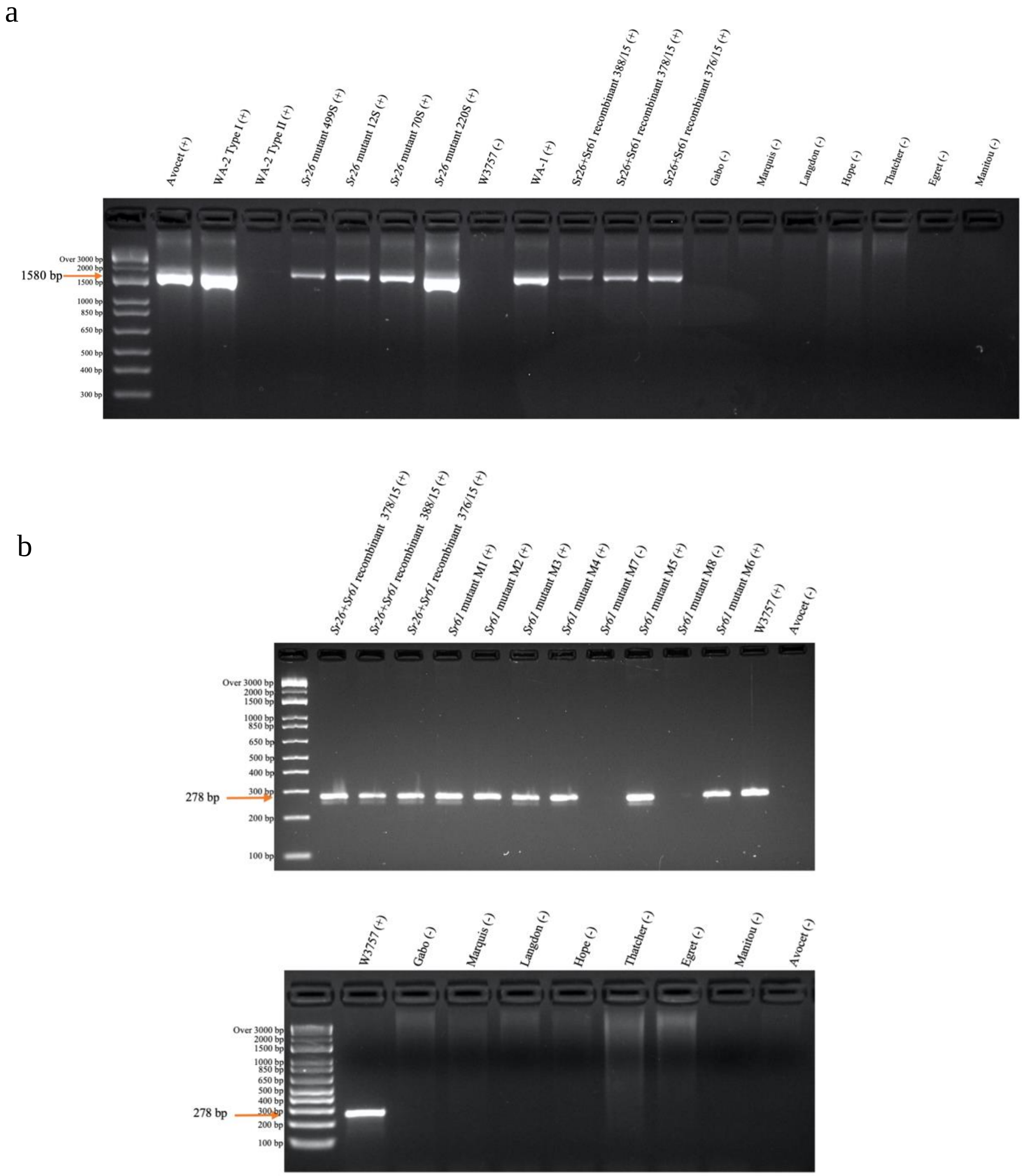

Supplementary Figure 4. Molecular marker analysis using *Sr26* and *Sr61* gene-specific markers on genetic stocks and mutant lines.

a. *Sr26* PCR marker analysis of selected plant genotypes. Genotype names are shown above the gel image and the transgene presence or absence were indicated as (+) or (-), respectively. Lines 378/15, 388/15 and 376/15 carry the same *Sr26*+*Sr61* recombinant chromosome. Lines WA-2 Type I and II are recombinants with and without *Sr26*, respectively. b. PCR marker analysis as described above except that an *Sr61*-specific marker was used. DNA derived from additional lines and cultivars were included as indicated. Each experiment was repeated independently at least 3 times with similar results. Source data are provided as Source Data file.

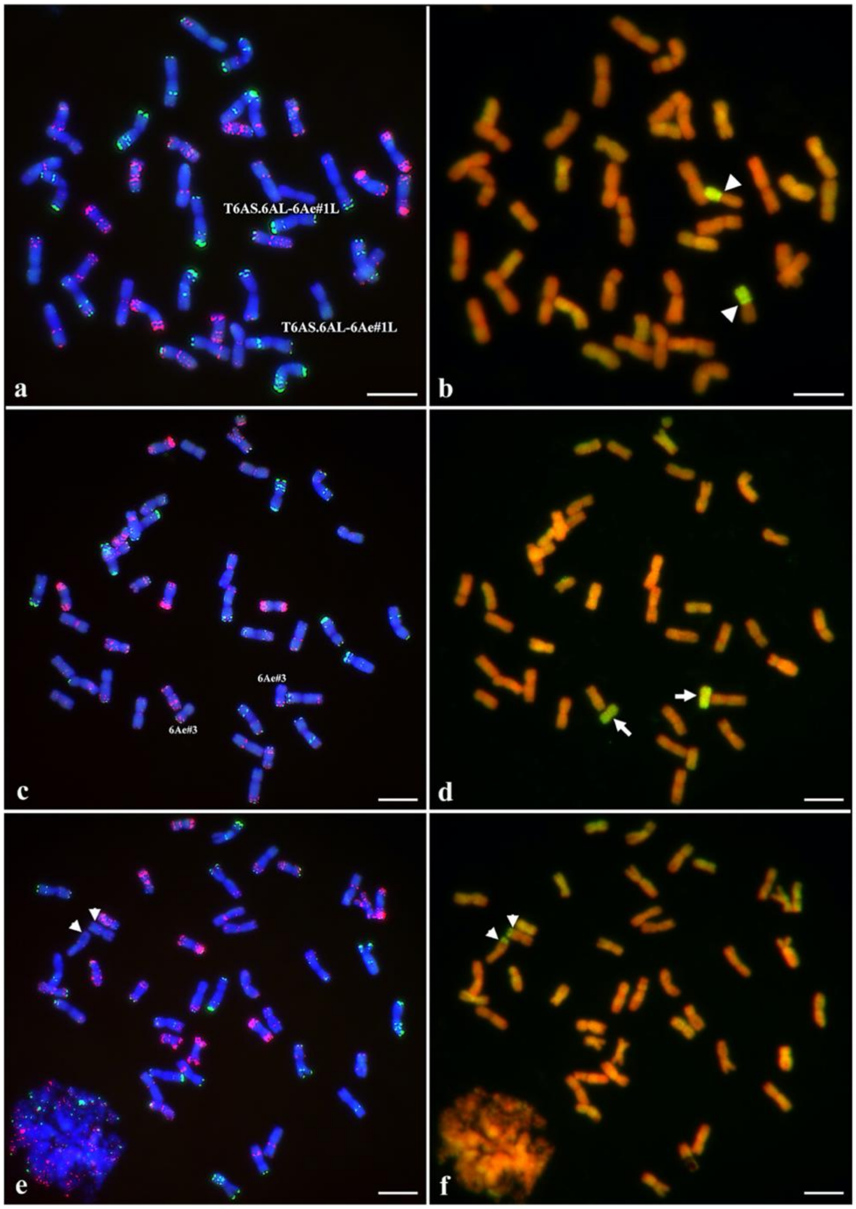

Supplementary Figure 5. Cytogenetic analysis of *Thinopyrum ponticum* 6Ae segments in wheat.

Panels (a), (c) and (e) are FISH images of metaphase chromosomes from Avocet+Lr46, W3757 and recombinant line 378/15, respectively. Chromosomes were hybridised with probe Oligo-pSc119.2-1 labeled with 6-carboxyfluorescein (6-FAM) and probe Oligo-pTa535-1 labelled with 6-carboxytetramethylrhodamine (Tamra) to generate green and red signals, respectively, which enabled the identification of individual chromosomes. Panels (b), (d), and (f) are identical chromosome spreads as in (a), (c), and (d), respectively, after GISH analysis was done. For FISH, chromosomes were counterstained with 4',6-diamidino-2-phenylindole (DAPI) and pseudo-colored blue. For GISH, *Pseudoroegneria stipifolia* DNA was labeled with biotin-16-dUTP and detected with fluorescein-avidin DN, which fluoresced yellow-green. Wheat chromosomes were counterstained with DAPI and pseudo-colored red with arrowheads (b, f) pointing to the translocation breakpoints of *Th. ponticum* (yellow) and wheat (red) chromosome segments and arrows pointing to the centromeres of *Th. ponticum* chromosomes (d). Bars show 10 µm. Each experiment was repeated independently at least 3 times with similar results.

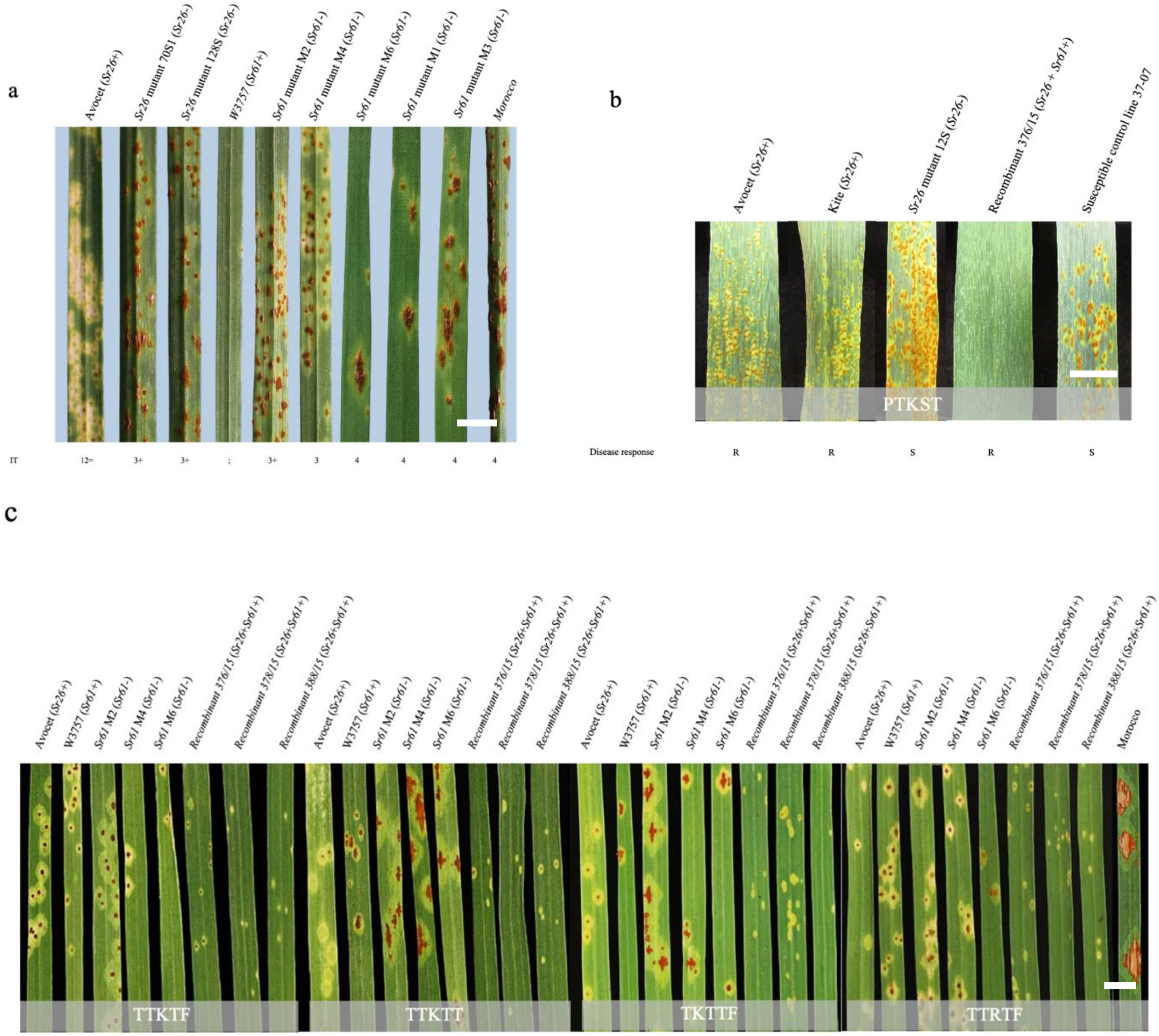

Supplementary Figure 6. Seedling and adult plant stem rust responses in lines containing *Sr26* and *Sr61* when infected with multiple *Pgt* races.

a. Stem rust responses of seedlings from selected *Sr26*, *Sr61* and mutant lines infected with *Pgt* 34-1,2,3,4,5,6,7 at 12 days post inoculation (dpi) under greenhouse conditions. Infection types scored on the 0-4 Stakman scale are indicated at the bottom. Bar shows 1 cm.

b. Flag leaf rust reactions to race *Pgt* PTKST at 14 dpi under greenhouse conditions. Recombinant line 376/15 which combines *Sr26* and *Sr61* is more resistant than lines containing *Sr26* alone. Bar shows 1 cm.

c. Stem rust responses of parent, mutant and recombinant seedlings at 12 dpi with *Pgt* races TTKTF, TTKTT, TKTTF, and TTRTF. Bar shows 1 cm.

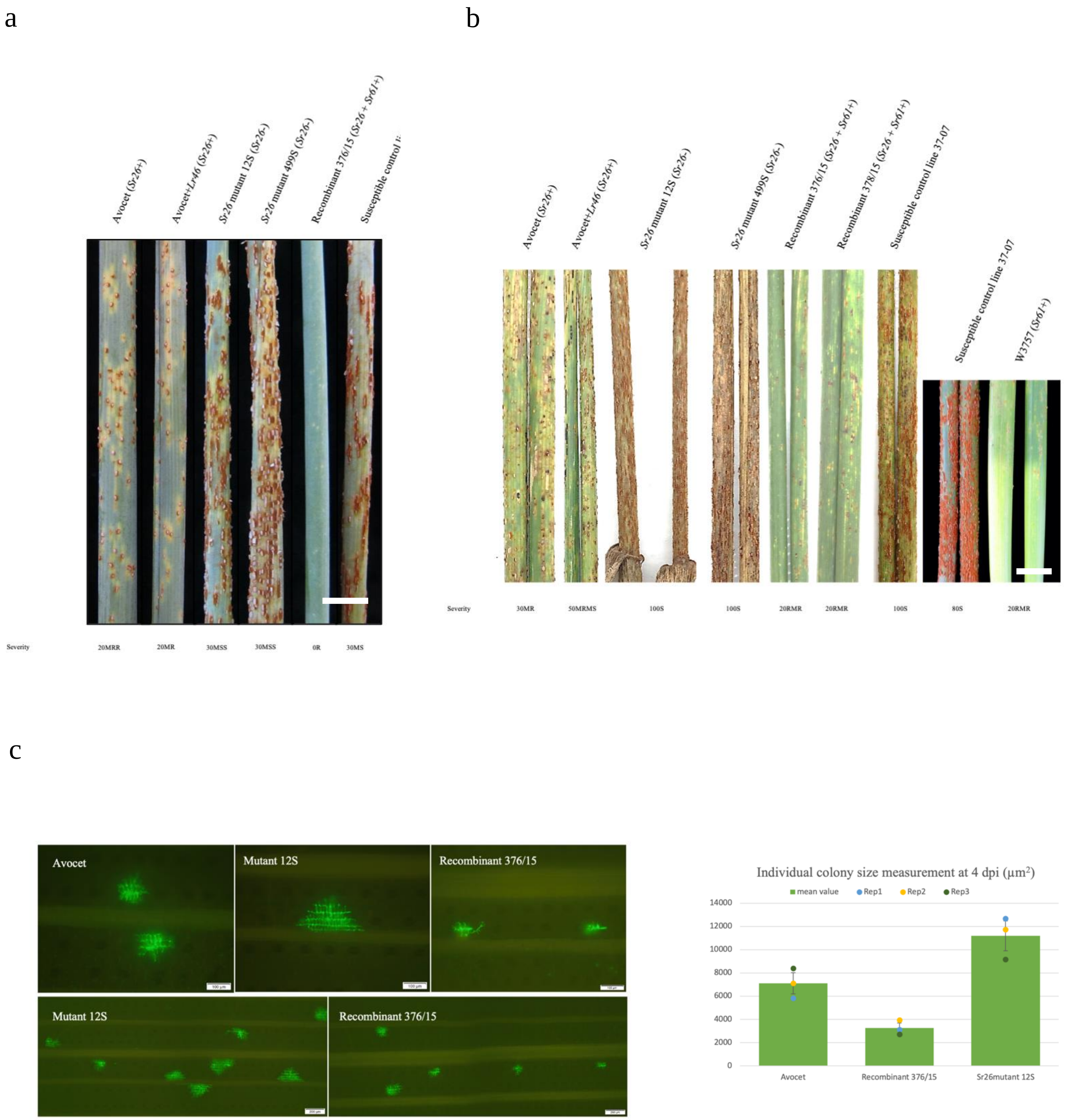
a
b
c
Avocet (Sr26+)
Avocet+Lr46 (Sr26+)
Sr26 mutant 12S (Sr26-)
Sr26 mutant 499S (Sr26-)
Recombinant 376/15 (Sr26 + Sr61+)
Susceptible control
Severity
20MRR
20MR
30MSS
30MSS
0R
30MS
Avocet (Sr26+)
Avocet+Lr46 (Sr26+)
Sr26 mutant 12S (Sr26-)
Sr26 mutant 499S (Sr26-)
Recombinant 376/15 (Sr26 + Sr61+)
Recombinant 378/15 (Sr26 + Sr61+)
Susceptible control line 37-07
Susceptible control line 37-07
W3757 (Sr61+)
Severity
30MR
50MRMS
100S
100S
20RMR
20RMR
100S
80S
20RMR
Avocet
Mutant 12S
Recombinant 376/15
Mutant 12S
Recombinant 376/15
Individual colony size measurement at 4 dpi (μm²)
mean value
Rep1
Rep2
Rep3
14000
12000
10000
8000
6000
4000
2000
0
Avocet
Recombinant 376/15
Sr26mutant 12S

d

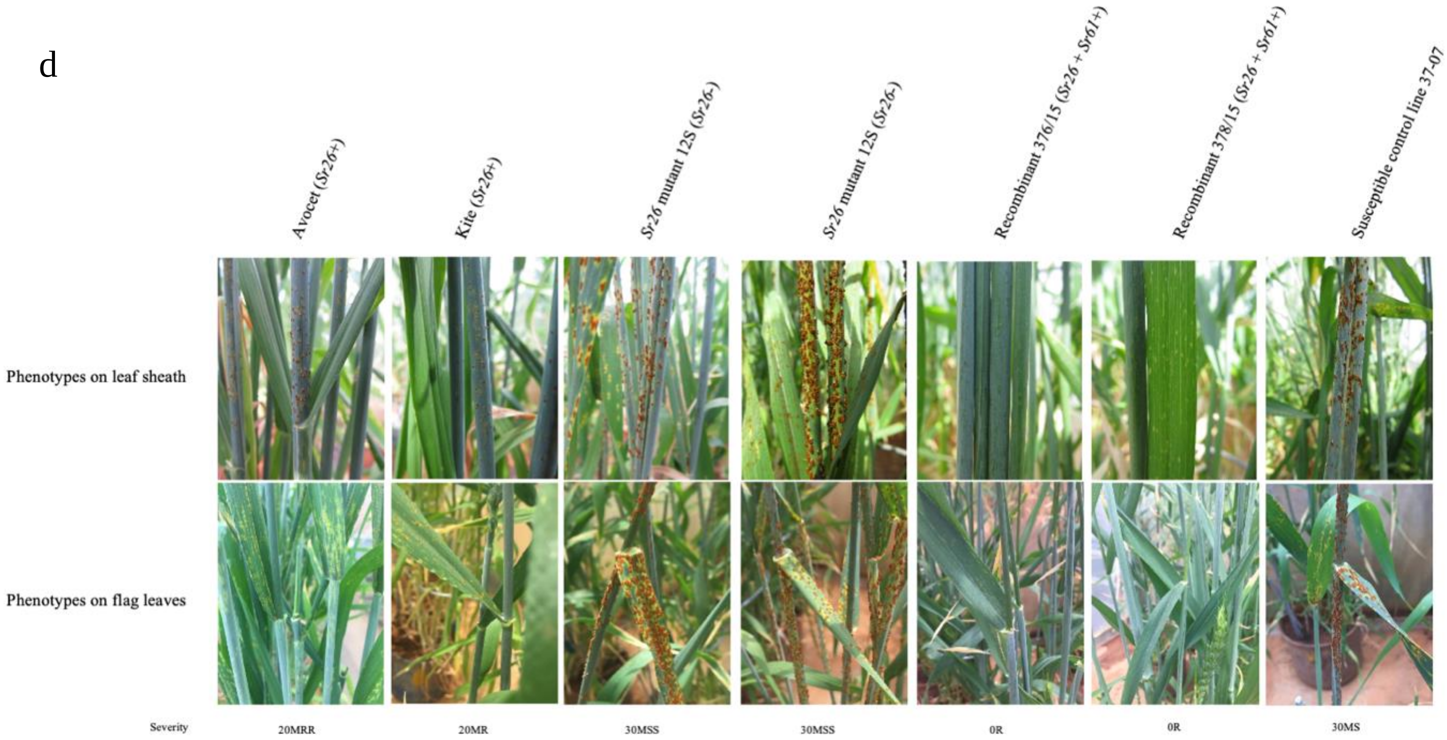

Supplementary Figure 7. Stem rust responses of leaf sheaths and stems infected with *Pgt* race PTKST.
a-b. *Pgt* rust infection responses at 20 dpi on (a) leaf sheaths of adult plants grown under greenhouse conditions and (b) adult plant stems under field conditions. Tissues were scored on a 0-100% infection area scale. Bar shows 1 cm.
c. Microscopic observations of *Pgt* race PTKST infection in greenhouse grown adult plant leaf sheaths at 4 dpi (left) and a graph of average rust infection site size on the same tissue (right). Mean value of 10 infection sites was represented by a dot point. Data are presented as mean values of 3 sets of 10 infection sites mean value +/- SD. Similar trends were obtained from 3 biologically independent leaf samples examined over 3 independent experiments for each line.
d. Adult plant responses on leaf sheaths and flag leaves of selected lines to *Pgt* race PTKST under greenhouse conditions. Disease severities based upon a 0-100% infection area scale are shown under each entry with each score based on three biological and technical replicates. R, resistant; MRR, moderately resistant/resistant; MR, moderately resistant; MS, moderately susceptible); MSS, moderately susceptible/susceptible. Scale bar was not provided as the objectives in these photos are multi-focused, but the diameters of the adult wheat stems in these photos are ranging from 0.4-0.9 cm approximately and could be used as references.
Source data underlying Supplementary Figure 7c are provided as Source Data file.

Supplementary Table 1. Summary of wild-type and EMS-derived *Sr26* and *Sr61* mutants used for MutRenSeq analysis.

Mutant	Line number	Response (*Pgt* race 34-1,2,3,4,5,6,7)			Genotyping result		Mutation Type
					(*Sr26* marker #43)		
		R sib (M2)	Mutant (M2)	Progeny test (M3)	R sib (M2)	Mutant (M2)	
12S	12S-1	R	S	S	+	+	Putative point mutation (*Sr26* marker retained)
70S	70S-1	R	S	S	+	+	
128S	128S-1	R	S	S	+	+	
499S	499S-1	R	S	S	+	+	
150S	150S-1	R	S	S	+	-	Putative deletion mutant (*Sr26* marker lost)

Mutant	Line number	Response (*Pgt* race 34-1,2,3,4,5,6,7)			Genotyping result		Mutation Type
					(*Sr61* marker MWG798)		
		R sib (M2)	Mutant (M2)	Progeny test (M3)	R sib (M2)	Mutant (M2)	
Mutant1	6421.4S	R	S	S	+	+	Putative point mutation (*Sr61* marker retained)
Mutant2	6802.4S	R	S	S	+	+	
Mutant3	7505.4S	R	S	S	+	+	
Mutant4	7150.4S	R	S	S	+	+	
Mutant5	5858.4S	R	S	S	+	+	
Mutant6	7521.4S	R	S	S	+	+	
Mutant7	6735.5S	R	S	S	+	-	Putative deletion mutant (*Sr61* marker lost)
Mutant8	6904.4S	R	S	S	+	-	

Supplementary Table 2. Stem rust responses of T_0 Fielder transgenics containing *Sr26* and *Sr61* gene constructs 12 dpi with *Pgt* race 98-1,2,3,5,6+Sr50.

construct	Transformation experiment No.	T_0 event	Infection type
Sr26:Sr22RE	PC225	1	1+2-2
Sr26:Sr22RE	PC225	2	122+
Sr26:Sr22RE	PC225	3	2-,2
Sr26:Sr22RE	PC225	4	;,2-

Sr26:Sr22RE	PC225	5	1+2-
Sr26:Sr22RE	PC225	6	;,1,2-
Sr26:Sr22RE	PC225	7	2
Sr26:Sr22RE	PC225	8	122+
Sr26:Sr22RE	PC225	9	1,2
Sr26:Sr22RE	PC225	10	2-,2
Sr26:Sr22RE	PC225	11	1+2-
Sr26:Sr22RE	PC225	12	;122+
Sr26:Sr22RE	PC225	12	;,1,2=
Sr26:Sr22RE	PC225	13	2
Sr26:Sr22RE	PC225	14	1+2
Sr26:Sr22RE	PC225	15	1,2-
Sr26:Sr22RE	PC225	16	;,1-,1,2-,2
Sr26:Sr22RE	PC225	17	;,2,2-
Sr26:Sr22RE	PC225	18	2-,2
Sr26:Sr22RE	PC225	19	1,2
Sr26:Sr22RE	PC225	20	12-
Sr26:Sr22RE	PC225	21	1+2
Sr26:Sr22RE	PC225	22	;,1,2
Non-transformed control	/	/	3+
Sr26:Sr33RE	PC226	1	2-,2
Sr26:Sr33RE	PC226	2	1+2
Sr26:Sr33RE	PC226	3	;,22-
Sr26:Sr33RE	PC226	4	;,2-
Sr26:Sr33RE	PC226	5	2
Sr26:Sr33RE	PC226	6	1,2-
Sr26:Sr33RE	PC226	7	;,1,2-
Sr26:Sr33RE	PC226	8	2+,3
Sr26:Sr33RE	PC226	9	2-,2
Sr26:Sr33RE	PC226	10	2,2-
Sr26:Sr33RE	PC226	11	2,2-
Sr26:Sr33RE	PC226	12	1,2-
Sr26:Sr33RE	PC226	13	1+22+
Sr26:Sr33RE	PC226	14	2,2+
Non-transformed control	/	/	3+
Sr26:NativeRE	PC253	1	2-
Sr26:NativeRE	PC253	2	2-
Sr26:NativeRE	PC253	3	2-
Sr26:NativeRE	PC253	4	2-
Sr26:NativeRE	PC253	5	2
Sr26:NativeRE	PC253	6	2=
Sr26:NativeRE	PC253	7	2=
Sr26:NativeRE	PC253	8	2=
Sr26:NativeRE	PC253	9	2=
Sr26:NativeRE	PC253	10	1=
Sr26:NativeRE	PC253	11	1=
Sr26:NativeRE	PC253	12	2=
Sr26:NativeRE	PC253	13	2=
Sr26:NativeRE	PC253	14	;
Sr26:NativeRE	PC253	15	0;
Sr26:NativeRE	PC253	16	0;
Sr26:NativeRE	PC253	17	2=
Sr26:NativeRE	PC253	18	1;
Sr26:NativeRE	PC253	19	1;
Sr26:NativeRE	PC253	20	;
Sr26:NativeRE	PC253	21	;1=
Non-transformed control	/	/	3+
Sr26:NativeRE	PC253	1	2-
Sr26:NativeRE	PC253	2	2-
Sr26:NativeRE	PC253	3	2-
Sr26:NativeRE	PC253	4	2-
Sr26:NativeRE	PC253	5	2
Sr26:NativeRE	PC253	6	2=
Sr26:NativeRE	PC253	7	2=
Sr26:NativeRE	PC253	8	2=
Sr26:NativeRE	PC253	9	2=
Sr26:NativeRE	PC253	10	1=
Sr26:NativeRE	PC253	11	1=
Sr26:NativeRE	PC253	12	2=
Sr26:NativeRE	PC253	13	2=
Sr26:NativeRE	PC253	14	;
Sr26:NativeRE	PC253	15	0;
Sr26:NativeRE	PC253	16	0;
Sr26:NativeRE	PC253	17	2=
Sr26:NativeRE	PC253	18	1;
Sr26:NativeRE	PC253	19	1;
Sr26:NativeRE	PC253	20	;
Sr26:NativeRE	PC253	21	;1=
Non-transformed control	/	/	3+
Sr61:Sr26RE	PC315	1	;1-
Sr61:Sr26RE	PC315	2	1+,2-
Sr61:Sr26RE	PC315	3	;1
Sr61:Sr26RE	PC315	4	;1=C
Sr61:Sr26RE	PC315	5	;1,2=
Sr61:Sr26RE	PC315	6	;1-,2
Sr61:Sr26RE	PC315	7	;1=C
Sr61:Sr26RE	PC315	8	;1=
Sr61:Sr26RE	PC315	9	;1=
Sr61:Sr26RE	PC315	10	1,2,2+
Sr61:Sr26RE	PC315	11	;1,2-
Sr61:Sr26RE	PC315	12	2-
Sr61:Sr26RE	PC315	13	;,1C
Sr61:Sr26RE	PC315	14	;,1-C
Non-transformed control	/	/	3+

Supplementary Table 3. *Sr26* and *Sr61* gene-specific, diagnostic primers and additional sequencing primers used in this study.

	Primer Name	Sequence (5' to 3')	Tm °C
Sr26 sequencing primers	Sr26Seq1R	TCGGAATCGTTCCCGTGAATTGAAGCTA	-
	Sr26Seq2R	ATGCTCAGGATAAGGCGTGGATGAATGAGGT	
	Sr26Seq3F	GGGGAGATCAAATCGCTCACTCAT	
	Sr26Seq4F	GTACAATTTCAGTTTTAACTTCTCATCCTTGAG	
	Sr26Seq5F	TACAGTATGAGCTGACCCAGCGG	
	Sr26Seq6F	GGATAGACAATGAAAAATGAGGA	
	Sr26Seq7F	GCTTTTCTTGATTTAAAATCATAGGATGT	
	Sr26Seq8R	GATATTATTGTCGCTTCCCTTAAAAAC	
	Sr26Seq9F	TTCCGAGGGTCATAGTCTCTGGC	
	Sr26Seq10R	TCTCCCACAAAAGGCCATGTACTTCTTTAATTCACAAG	
Sr26 gene specific primers (diagnostic primers)	Sr26GSPF	GGAATACTCGAATACCAGGCCAT	58
	Sr26GSPR	TTGCCACTGTGAACATGTTTATAGAT	
Sr61 sequencing primers	Sr61Seq1	GCAGGTAACTCACAAGCATAACTAGGAG	-
	Sr61Seq2	GCCAATGAGGTGTACCATATG	
	Sr61Seq3	ATGCACTAAAGGTAGATCCTGG	
	Sr61Seq4	ATTATAATCAAGTACCTGCCAACATT	
	Sr61Seq5	ACAAAAGGAAAGGTGGAAGG	
	Sr61Seq6	GACGAGCCTTGTAATCCAA	
	Sr61Seq7	CGATATCTACGTGCATTTGATTTACG	
	Sr61Seq8	AACCAACAATTCGATGACACAAGG	
	Sr61Seq9	CAGACTCTGCCCATTCCGT	
	Sr61Seq10	TGCACATACTAGCCGCTTGATATTT	
Sr61 gene specific primers (diagnostic primers)	Sr61GSPF	AACCAACAATTCGATGACACAAGG	62
	Sr61GSPR	CGATATCTACGTGCATTTGATTTACG	

Supplementary Table 4. Homologue or best hit (E-value=0, query cover > 60%, identity >80%) of *Sr26* and *Sr61* in the genome assembly of *Thinopyrum elongatum* isolate D-3458 chromosome 6E, sequence ID CM022302.1 and in the genome assembly of CS Ref. V2.0.

Sequence query	Query cover	Percentage of identity	Homologue location	Genome assembly
Sr26 CDS with intron	89%	89.94	563037027-563035259	*Thinopyrum elongatum* isolate D-3458

Sr26 CDS	97%	90.20	563036881-563035259	
Sr61 CDS with intron	61%	83.98	29030858-29030041	
Sr61 CDS	69%	83.94	29030858-29030061	

Description	Identities (Query length)	Percentage of identity	Homologue location	Genome assembly
Sr26 CDS with intron	2418/3009 (6066)	80	717368007-717365128 (6B)	CS Ref. V2.0
Sr26 CDS	1727/1971 (2808)	88	717330560-717328588 (6B)	
Sr61 CDS with intron	1805/2335 (3098)	77	6514418394-651420682 (2D)	
Sr61 CDS	858/1058 (2643)	81	190178439-190179496(7D)	

Supplementary Table 5. Pathogenicity analysis of wild-type *Sr26* and *Sr61* lines and mutants.

Pgt Race	Avocet+ Lr46	*Sr26* Mutant 12S/70S	*Sr26* Mutant 128S	*Sr26* Mutant 499S	*Sr26* Mutant 150S	W3757	M1	M2	M3	M4	M5	M6	M7	M8	Recombinant 376/15	Recombinant 378/15	Recombinant 388/15
34-1,2,3,4,5,6,7	R	S	S	S	S	R	S	S	S	S	S	S	S	S	R	R	R
98-1,2,3,5,6+Sr50	R	S	S	S	S	R	S	S	S	S	S	S	N/D	N/D	R	R	R
21-0	R	S	S	S	S	R	S	S	S	S	S	S	N/D	N/D	R	R	R
PTKST	R	S	N/D	S	N/D	R	N/D	S	N/D	S	N/D	S	N/D	N/D	R	R	N/D
TTKTT	R	S	N/D	N/D	N/D	R	N/D	S	N/D	S	N/D	S	N/D	N/D	R	R	R
TTRTF	R	S	N/D	S	N/D	R	N/D	R	N/D	R	N/D	R	N/D	N/D	R	R	R
TTKTF	R	N/D	N/D	N/D	N/D	R	N/D	R	N/D	R	N/D	R	N/D	N/D	R	R	R
TKTTF	R	N/D	N/D	N/D	N/D	R	N/D	R	N/D	R	N/D	R	N/D	N/D	R	R	R

Supplementary Table 6. Summary of *Pgt* races used in this study.

Race name	Isolate accession number	Location of origin	Location of experiment undertaken
Pgt 21-0	330	Australia	Plant breeding institute, The University of Sydney, Australia
Pgt 34-1,2,3,4,5,6,7	107	Australia	Plant breeding institute, The University of Sydney, Australia
Pgt 98-1,2,3,5,6+Sr50	632	Australia	Plant breeding institute, The University of Sydney, Australia; CSIRO Black Mountain Laboratory, Canberra, Australia
Pgt PTKST	UVPgt60#2/13/10/2010	South Africa	University of the Free State, Bloemfontein, South Africa
TTKTT	KE178b/18	Kenya	Aarhus Unversity, Flakkebjerg, Slagelse, Denmark
TTRTF	IT189a/18	Italy	Aarhus Unversity, Flakkebjerg, Slagelse, Denmark
TTKTF	LB37a/12	Lebanon	Aarhus Unversity, Flakkebjerg, Slagelse, Denmark
TKTTF	DK185a/13	Denmark	Aarhus Unversity, Flakkebjerg, Slagelse, Denmark
