# Supplementary material for: A recombined Sr26 and Sr61 disease resistance gene stack in wheat encodes unrelated NLR genes
Source: Nat Commun. 2021 Jun 7;12:3378. doi: 10.1038/s41467-021-23738-0 (PMC8184838; doi:10.1038/s41467-021-23738-0)
Supplement: Supplementary file 5 — Description of Additional Supplementary Files [file 41467_2021_23738_MOESM5_ESM.pdf]

Description of additional supplementary files

Title: Supplementary Data 1
Description: Proteins used for phylogenetic tree construction. All 123 proteins used in constructing the phylogenetic tree were listed in the table with their *R* gene names, source organisms, pathogens/diseases and the protein types.

Title: Supplementary Data 2
Description: Summary of MutRenSeq NGS data of wildtype *Sr26* and *Sr61* lines and mutants. Detailed information of the NGS data used for identifying *Sr26* and *Sr61* were showing in the table. Raw reads: four rows are taken as a unit to calculate the total amount of read1 and read2 in raw data files; Raw bases: (total raw reads) * (sequence length), calculating in G; Error rate: base error rate; Q20, Q30: (Base count of Phred value > 20 or 30) / (Total base count) GC content: (G & C base count) / (Total base count).
