# Supplementary material for: A recombined Sr26 and Sr61 disease resistance gene stack in wheat encodes unrelated NLR genes
Source: Nat Commun. 2021 Jun 7;12:3378. doi: 10.1038/s41467-021-23738-0 (PMC8184838; doi:10.1038/s41467-021-23738-0)
Supplement: Supplementary file 6 — Reporting Summary [file 41467_2021_23738_MOESM6_ESM.pdf]

nature research

Corresponding author(s): Evans Lagudah, Peng Zhang

Last updated by author(s): Apr 28, 2021

Reporting Summary

Nature Research wishes to improve the reproducibility of the work that we publish. This form provides structure for consistency and transparency in reporting. For further information on Nature Research policies, see our Editorial Policies and the Editorial Policy Checklist.

Statistics

For all statistical analyses, confirm that the following items are present in the figure legend, table legend, main text, or Methods section.

n/a	Confirmed	
☐	☒	The exact sample size (n) for each experimental group/condition, given as a discrete number and unit of measurement
☐	☒	A statement on whether measurements were taken from distinct samples or whether the same sample was measured repeatedly
☒	☐	The statistical test(s) used AND whether they are one- or two-sided<br>*Only common tests should be described solely by name; describe more complex techniques in the Methods section.*
☒	☐	A description of all covariates tested
☒	☐	A description of any assumptions or corrections, such as tests of normality and adjustment for multiple comparisons
☐	☒	A full description of the statistical parameters including central tendency (e.g. means) or other basic estimates (e.g. regression coefficient) AND variation (e.g. standard deviation) or associated estimates of uncertainty (e.g. confidence intervals)
☒	☐	For null hypothesis testing, the test statistic (e.g. F, t, r) with confidence intervals, effect sizes, degrees of freedom and P value noted<br>*Give P values as exact values whenever suitable.*
☒	☐	For Bayesian analysis, information on the choice of priors and Markov chain Monte Carlo settings
☒	☐	For hierarchical and complex designs, identification of the appropriate level for tests and full reporting of outcomes
☒	☐	Estimates of effect sizes (e.g. Cohen's d, Pearson's r), indicating how they were calculated

Our web collection on statistics for biologists contains articles on many of the points above.

Software and code

Policy information about availability of computer code

Data collection	No software was used for data collection
Data analysis	CLC Genomics Workbench v9.0 (Sr26) and v10.0 (Sr61) (Qiagen, Hilden, Germany) (https://digitalinsights.qiagen.com/products-overview/discovery-insights-portfolio/analysis-and-visualization/qiagen-clc-genomics-workbench/)<br>MutantHunter pipeline (https://github.com/steuernb)<br>MuTrigo Python package (https://github.com/TC-Hewitt/MuTrigo)<br>MYbaits protocol and the Triticeae NLR bait libraries (https://github.com/steuernb/MutantHunter/blob/master/Triticea_RenSeq_Baits_V3. fasta.gz)<br>MEGA X (MUSCLE and UPGMA)(https://www.megasoftware.net) v10<br>ITOL (https://itol.embl.de) v6<br>COILS prediction program (https://embnet. vital-it.ch/software/COILS_form.html) v2.2<br>T-Coffee program (http://tcoffee.crg.cat/apps/tcoffee/do:expresso) version:Expresso<br>AnalySIS LS Research v2.2<br>Photoshop vCS6

For manuscripts utilizing custom algorithms or software that are central to the research but not yet described in published literature, software must be made available to editors and reviewers. We strongly encourage code deposition in a community repository (e.g. GitHub). See the Nature Research guidelines for submitting code & software for further information.

Data

Policy information about availability of data

All manuscripts must include a data availability statement. This statement should provide the following information, where applicable:

- Accession codes, unique identifiers, or web links for publicly available datasets
- A list of figures that have associated raw data
- A description of any restrictions on data availability

Annotated genomic sequences of Sr26 and Sr61 have been deposited at NCBI Gen Bank with accession numbers MN531843 (Sr26) and MN531844 (Sr61); All protein accession numbers listed in Supplementary Figure S7 are publicly available at NCBI Genbank (https://www.ncbi.nlm.nih.gov/).

Field-specific reporting

Please select the one below that is the best fit for your research. If you are not sure, read the appropriate sections before making your selection.

☒ Life sciences ☐ Behavioural & social sciences ☐ Ecological, evolutionary & environmental sciences

For a reference copy of the document with all sections, see nature.com/documents/nr-reporting-summary-flat.pdf

Life sciences study design

All studies must disclose on these points even when the disclosure is negative.

Sample size	All the information regarding sample size were specifically described in the manuscript
Data exclusions	No data were excluded from the analyses.
Replication	Mutant plants were confirmed by at least three rounds of independent phenotyping across three generations
Randomization	When phenotyping plants, we randomized mutant and wild-type controls as is normal practice.<br>It is not relevant to other experiments as no subjects were allocated to experimental groups.
Blinding	Blinding was performed when phenotyping the mutant lines for their resistance to Pgt, candidate gene confirmation by wheat transformation, rust phenotyping and hstological assemssement, characterisation of T-DNA copy umber by Digital PCR, qRT-PCR for determining transgene expression. It is not performed when carrying out RenSeq, Sequencing analysis, phylogenetic analysis, protein structure predictions, and molecular cytogenetic characterisation of lines containing Sr26 and Sr61 as required by the experiments.

Reporting for specific materials, systems and methods

We require information from authors about some types of materials, experimental systems and methods used in many studies. Here, indicate whether each material, system or method listed is relevant to your study. If you are not sure if a list item applies to your research, read the appropriate section before selecting a response.

Materials & experimental systems

n/a	Involved in the study
☒	☐ Antibodies
☒	☐ Eukaryotic cell lines
☒	☐ Palaeontology and archaeology
☒	☐ Animals and other organisms
☒	☐ Human research participants
☒	☐ Clinical data
☒	☐ Dual use research of concern

Methods

n/a	Involved in the study
☒	☐ ChIP-seq
☒	☐ Flow cytometry
☒	☐ MRI-based neuroimaging
